# The Gastrointestinal Load of Carbapenem-Resistant Enterobacteriacea Is Associated With the Transition From Colonization to Infection by *Klebsiella pneumoniae* Isolates Harboring the *bla_KPC_* Gene

**DOI:** 10.3389/fcimb.2022.928578

**Published:** 2022-07-05

**Authors:** Letícia Busato Migliorini, Laura Leaden, Romário Oliveira de Sales, Nathalia Pellegrini Correa, Maryana Mara Marins, Paula Célia Mariko Koga, Alexandra do Rosario Toniolo, Fernando Gatti de Menezes, Marines Dalla Valle Martino, Jesús Mingorance, Patricia Severino

**Affiliations:** ^1^ Albert Einstein Research and Education Institute, Hospital Israelita Albert Einstein, Sao Paulo, Brazil; ^2^ Laboratório Clínico, Hospital Israelita Albert Einstein, Sao Paulo, Brazil; ^3^ Serviço de Controle de Infecção Hospitalar, Hospital Israelita Albert Einstein, Sao Paulo, Brazil; ^4^ Servicio de Microbiología, Hospital Universitario La Paz, IdiPAZ, Madrid, Spain

**Keywords:** gastrointestinal carriage, *Klebsiella pneumoniae*, bla KPC gene, Carbapenem-resistant Enterobacteriaceae (CRE), virulence factors

## Abstract

**Background:**

Healthcare-associated infections by carbapenem-resistant *Klebsiella pneumoniae* are difficult to control. Virulence and antibiotic resistance genes contribute to infection, but the mechanisms associated with the transition from colonization to infection remain unclear.

**Objective:**

We investigated the transition from carriage to infection by *K. pneumoniae* isolates carrying the *K. pneumoniae* carbapenemase–encoding gene *bla*_KPC_ (KpKPC).

**Methods:**

KpKPC isolates detected within a 10-year period in a single tertiary-care hospital were characterized by pulsed-field gel electrophoresis (PFGE), multilocus sequencing typing, capsular lipopolysaccharide and polysaccharide typing, antimicrobial susceptibility profiles, and the presence of virulence genes. The gastrointestinal load of carbapenem-resistant Enterobacteriaceae and of *bla*_KPC_-carrying bacteria was estimated by relative quantification in rectal swabs. Results were evaluated as contributors to the progression from carriage to infection.

**Results:**

No PGFE type; ST-, K-, or O-serotypes; antimicrobial susceptibility profiles; or the presence of virulence markers, such yersiniabactin and colibactin, were associated with carriage or infection, with ST437 and ST11 being the most prevalent clones. Admission to intensive and semi-intensive care units was a risk factor for the development of infections (OR 2.79, 95% CI 1.375 to 5.687, *P*=0.005), but higher intestinal loads of carbapenem-resistant Enterobacteriaceae or of *bla*_KPC_-carrying bacteria were the only factors associated with the transition from colonization to infection in this cohort (OR 8.601, 95% CI 2.44 to 30.352, *P*<0.001).

**Conclusion:**

The presence of resistance and virulence mechanisms were not associated with progression from colonization to infection, while intestinal colonization by carbapenem-resistant Enterobacteriacea and, more specifically, the load of gastrointestinal carriage emerged as an important determinant of infection.

## Introduction

*Klebsiella pneumoniae* is an important microorganism in healthcare-associated infections and also known as a primary dissemination source of resistance mechanisms, among which are *K. pneumoniae* carbapenemase (KPC) and New Delhi metalobetalactamase (NDM) (Nordmann et al., 2009; Nordmann et al., 2011; Sampaio and Gales, 2016).

The spread of multidrug (MDR) and hypervirulent (Hv) *K. pneumoniae* lineages impacts mortality rates, reaching 95% for Hv strains, and 42% *versus* 21% for MDR and susceptible strains, respectively (Xu et al., 2017; Andrey et al., 2020). These lineages may be associated with specific sequence types (STs), such as ST16, ST11, ST437, ST258, and ST512 (Andrey et al., 2020). In Brazil, KPC-carrying *K. pneumoniae* (KpKPC), mainly ST437 and ST11, are epidemic and considered a public health threat (Nicoletti et al., 2012; Sampaio and Gales, 2016; Andrey et al., 2020; Migliorini et al., 2021). Due to the restricted antibiotic options to treat KpKPC infections, identifying the risks and preventing infections are considered a promising therapeutic approach.

Gastrointestinal carriage contributes to the spread of *K. pneumoniae* lineages in the hospital environment and to the development of infections (Lerner et al., 2015; Joseph et al., 2021; Lázaro-Perona et al., 2021; Falcone et al., 2022). Although virulence factors are necessary for the establishment of infections, they alone do not explain the progression from colonization to infection as recently reported (Joseph et al., 2021). For instance, siderophore yersiniobactin, encoded by *ybt* genes, are commonly present in strains involved with infections but their presence does not explain this progression (Joseph et al., 2021). Recently, the extent of intestinal colonization has emerged as a risk factor for the transition from colonization to infection (Tacconelli et al., 2019; Ramos-Ramos et al., 2020).

Aiming to investigate the transition from intestinal colonization by Carbapenem-resistant Enterobacteriaceae (CRE) to infection by carbapenem-resistant KpKPC, we characterized epidemiologically unrelated KpKPC carriage and infection isolates detected within a 10-year period in a single tertiary hospital and results were interpreted together with the intestinal load of CRE and of *bla*_KPC_-carrying bacteria.

## Materials and Methods

### Isolates and Clinical Samples

Between 2011 and 2021, 651 patients admitted to the semi- and intensive care units were carriers of KpKPC according to rectal swab analysis (carriage isolates). Infection isolates were those obtained from blood, bronchial lavage, ascitic fluid, abdomen secretion, tracheal secretion, and urine, and out of the 651 carriers, 128 developed a KpKPC infection after 30 days of carriage detection (i.e., positive rectal swab sample for KpKPC). The viable bacterial samples that could be reactivated in culture media and corresponded to the first KpKPC-positive carriage and/or infection sample of non-repeated patients were considered for the study. Considering these criteria, both carriage and infection isolates from 54 patients who had infection following colonization (n = 108 isolates) and 68 carriers (n = 68 isolates) were selected. A total of 176 isolates from 122 different patients were evaluated in this study. All 176 isolates were characterized by PFGE, and 124 were sequenced, corresponding to at least one isolate per patient included in the study. For patients who developed an infection associated with the same KpKPC isolate according to PFGE analyses (≥90% similarity), only the infection isolate was sequenced.

For the relative quantification of the intestinal load, rectal swabs from KpKPC carriers admitted to the hospital between June 2020 and December 2021 were selected. In this period, 165 patients were KpKPC carriers, from which 58 developed an infection associated with the presence of KpKPC within 30 days of the KpKPC-positive rectal swab. Only one sample per patient was included in the study, corresponding to the first KpKPC-positive rectal swab.

This study was approved by the Institutional Review Board (CAAE: 98373618.0.0000.0071).

### Microbiological Analysis

*K. pneumoniae* isolates were identified by Matrix Assisted Laser Desorption Ionization Time Of Flight Mass Spectrometry (MALDI-TOF MS) (Bruker Daltonics, Billerica, MA, USA). Antibiotic susceptibility was determined using the Vitek 2 XL System (bioMérieux, Craponne, France) and confirmed by the epsilometric (Etest^®^) method for carbapenems (imipenem or meropenem). Antimicrobial susceptibility results were interpreted according to the most recent Brazilian Committee on Antimicrobial Susceptibility Testing/European Committee on Antimicrobial Susceptibility Testing (BrCAST/EUCAST) guidelines (BrCAST, 2020). Carriage isolates were tested for imipenem and meropenem (n = 122). Infection isolates (n = 54) were tested for imipenem and meropenem and also for cephalosporins (ceftazidime and cefepime), aminoglycosides (amikacin and gentamicin), and ciprofloxacin.

### Molecular Detection of the *bla*_KPC_ Gene

Real-time PCR was used to detect the *bla*_KPC_ gene in isolates showing reduced susceptibility to carbapenem. For this protocol, DNA extraction was performed using the PrepMan™ kit (Thermo Fisher Scientific, Waltham, Massachusetts, United States). DNA (10 ng) was added to the TaqMan master mix containing specific probes for *bla*_KPC_ (FAM fluorophore – 5´ TG ATA ACG CCG CCG CCA ATT TGT 3´) and 16S rRNA (CY5 fluorophore – 5´ CA CGA GCT GAC GAC AR*C CAT GCA 3’), as well as specific primers for *bla*_KPC_ (Forward: 5´ GGCCGCCGTGCAATAC 3´ and Reverse: 5’ GCCGCCCAACTCCTTCA 3’) and 16S rRNA (Forward: 5’ TGGAGCATGTGGTTTAATTCGA 3’ and Reverse: 5’ TGCGGGACTTAACCCAACA 3’). The mixture was inserted in a microfluidic cartridge and processed by the BD MAX™ equipment using the PCR Only module. Results were released qualitatively: the presence or absence of the gene.

### Pulsed-Field Gel Electrophoresis

The genetic relatedness was established by pulsed-field gel electrophoresis (PFGE) as previously described (Han et al., 2013). For the PFGE result interpretation, the Dice similarity coefficient was used and isolates were considered identical when patterns showed ≥ 90% similarity (Koroglu et al., 2015).

### Whole Genome Sequencing

Genomic DNA isolation for whole-genome sequencing (WGS) was performed as previously described (Salvà Serra et al., 2018). The concentration and purity of genomic DNA was assessed with a NanoDrop™ One Spectrophotometer (Thermo Fisher Scientific). DNA fragmentation for library construction was performed using the Ion Shear Plus reagents kit, and libraries were constructed using the Ion Plus Fragment Library Kit (Thermo Fisher Scientific). Barcoded libraries were quantified using the Bioanalyzer 2100 and High Sensitivity DNA kit (Agilent, Santa Clara, California, United States). Clonal amplification of the libraries was carried out using Ion PI™ Hi-Q™ Chef Kit (Thermo Fisher Scientific) and sequenced using the Ion PI™ Chip Kit v3 and Ion PI™ Hi-Q™ Sequencing 200 Kit (Thermo Fisher Scientific) in the Ion Proton Sequencer. Quality filtering was done with cutadapt v3.4 (Martin, 2011). Assembly was performed with SPAdes genome assembler software (v3.15.0) using the "iontorrent" and "careful" options (Prjibelski et al., 2020).

### Multilocus Sequencing Typing, O:K-Typing, Resistance, and Virulence Gene Analysis

MLST, the detection of virulence and antibiotic resistance genes, and lipopolysaccharide (O) and capsular polysaccharide (K) characterization were based on the analysis of WGS data. Sequence types (STs) were determined using the multilocus sequencing typing (MLST) *script* available at https://github.com/tseemann/mlst implemented at Institut Pasteur (Diancourt et al., 2005). Resistance and virulence genes were identified using the ABricate tool available at https://github.com/tseemann/abricate. ABricate allows the screening of contigs against multiple databases, including the AMRFinderPlus resistance gene database (Feldgarden et al., 2019) and the Virulence Factor Database (VFDB) (Chen et al., 2005).

### Relative Quantification of the Gastrointestinal Load of CRE

Rectal swabs were eluted in 0.5 mL of the TE 1X buffer (Tris-EDTA; 10 mM Tris, 1mM EDTA, pH 8.0) and used for serial 10-fold dilution in 0.9% saline as previously described (Lerner et al., 2013). The dilutions were plated on tryptic soy plates supplemented with 5% sheep blood (BiobioMérieux, Craponne, France) and CHROMagar ™ msupercarba™ plates (CHROMagar, Paris, France), to obtain total viable aerobic bacteria (TAB) and viable CRE, respectively. Bacterial counts were determined after 18 h of growth at 37°C, and the ratio of CRE to TAB [colony-forming unit (CFU/ml)] was determined and expressed as log CRE/TAB. The rectal swab suspensions were stored at -20°C for further molecular analysis.

### Relative Quantification of the Gastrointestinal Load of *bla*_KPC_ Gene

Total DNA was extracted from rectal swab suspensions. Briefly, 250 µl of bacterial suspensions were heated at 100°C for 10 min and centrifuged at 12,000*g* for 5 min, and the lysate was transferred to a new tube. Quantitative real-time PCR was performed in multiplexed reactions using primers for the 16S rRNA gene (see above) and following conditions previously described (Centers for Disease Control and Prevention (CDC), 2019). DNA from a *K. pneumoniae* isolate harboring the *bla*_KPC_ gene, Kp378, previously sequenced by our group (Migliorini et al., 2021) was used as positive control. The *bla*_KPC_ copy number of the positive control (Kp378) was determined from short-read assemblies by dividing the coverage of the contig containing *bla*_KPC_ by the average coverage for the assembly (weighted by contig length) as previously described (Stoesser et al., 2020). The 2^-ΔΔCt^ method was used to compare *bla*_KPC_ copy numbers between the rectal swab and the reference samples (Ramos-Ramos et al., 2020) ΔΔCt was defined as the difference between the ΔCt of a sample (rectal swab) and ΔCt of the reference sample (Kp378). Relative loads (RLs) were defined as RL= log(2^-ΔΔCt^), in which values close to zero meant that the sample contained a similar copy number of *bla*_KPC_ when compared to the reference sample, and values lower or higher than zero were considered as low or high intestinal loads of bacteria carrying *bla*_KPC_ gene, respectively.

### Statistical Analysis

Electronic clinical records were searched retrospectively. The variables registered were sex, age, comorbidities, hospitalization days within last 2 months, medical ward admission, antibiotic treatments in the previous 30 days, invasive procedures, and infections. The statistical significance of results was tested by non-parametric Mann–Whitney U test and two-way ANOVA for continuous variables and Fisher’s exact test for categorical variables (Graph Pad Software 9.0 Inc). Bivariate analysis to determine the impact of covariables on infection following colonization was carried out by binary logistic regression, adjusting for confounders, using jamovi (Version 1.6).

## Results

### Molecular Characterization of Carriage and Infection Isolates

Out of 122 KpKPC carrier patients from which isolates were selected for this study, 54 had a subsequent infection episode; from these 54 cases, both carriage and infection KpKPC isolates were analyzed by PFGE (n = 108). In 46 of these cases (85%) the same KpKPC strain was detected as carriage and in the infection site (≥90% similarity in the PFGE pattern), suggesting that KpKPC intestinal colonization was the primary source for infection.

**Figure 1:**
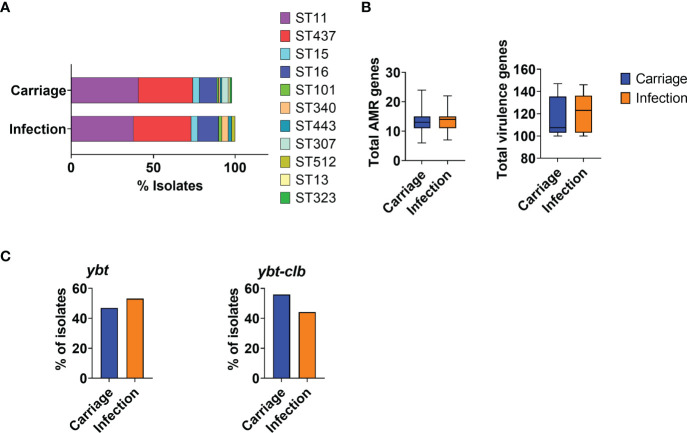
Characterization of KpKPC isolates in carriage and infection groups according to MLST and acquired resistance and virulence genes. **(A)** Bar graph showing the distribution of KpKPC isolates STs in carriage and infection groups. The most prevalent STs were ST11 and ST437 in both groups. **(B)** Total number of acquired resistance and virulence genes in carriage and infection groups. **(C)** Total number of isolates carrying the yersiniobactin (*ytb*) and colibactin (*clb*) *loci*. Both groups (carriage and infection) showed a similar number of acquired genes associated with antimicrobial resistance and virulence.

At least one isolate per patient was sequenced; general genome data quality, STs, O-, and K-types are reported in **Supplementary Table 1**. Most carriage and infection isolates belonged to the most prevalent strains in the period, ST11 or ST437, and no specific ST could be associated with either carriage or infection in this cohort (**Figure 1A**).

Additionally, capsule polysaccharide synthesis and O antigen *locus* analysis indicated that isolates belonged to 15 distinct K-*loci* groups, the most recurrent being KL36, corresponding to ST437. We also identified a total of four different O-types, of which O4 was predominant. No differences between carriage and infection samples were observed.

### Antimicrobial Susceptibility, and Virulence Genes

All included isolates were resistant to carbapenems (imipenem or meropenem). Among infection isolates, resistance rates were high for ciprofloxacin (98%), ceftazidime (93%), and cefepime (93%) and lower for amikacin (48%) and gentamicin (44%). The *bla*_KPC-2_ gene was the most frequently found gene encoding a carbapenamase (102/124), followed by *bla*_KPC-3_ (3/124), *bla*_KPC-30_ (2/124), and *bla*_KPC-33_ (1/124). One isolate of *K. pneumoniae* carried *bla*_NDM_. No other carbapenem resistance mechanisms were found. Additionally, genes associated with resistance to cephalosporins (*bla*_CTX-M-15_, *bla*_OXA-1_, *bla*_TEM-1_, and *bla*_SHV-158_) and to aminoglycoside (*aac(6’)-Ib*-*D181Y*) were also detected, as well as genes associated with reduced susceptibility to ciprofloxacin (*oqxA* and *oqxB*). All infection-associated isolates resistant to ciprofloxacin, cephalosporins, and/or aminoglycoside carried genes related to resistance to these antibiotic classes, showing a good association between the resistance phenotype and genetic determinants. The number of resistance genes was not significantly altered over time, and no significant differences were observed between carriage isolates and those related to infection (**Figure 1B**). Surprisingly, virulence genes were not mostly found in the isolates involved with infection nor did the number of virulence genes vary (**Figure 1B**). Carriage and infection isolates also carried a very similar set of virulence markers (data not shown), including yersiniobactin (*ytb*) and colibactin (*clb*) (**Figure 1C**).

Taken together, these results indicate that the resistance and virulence markers identified in this study, or their combination, possibly contribute but do not explain infections identified following intestinal colonization by KpKPC.

### Intestinal Carriage of CRE and *bla*_KPC_-Carrying Bacteria in Colonized Patients

The relative intestinal load of CRE and *bla*_KPC_-carrying bacteria (bacKPC) was estimated by microbiological and molecular quantification methods, respectively. Out of the 165 patients evaluated, 58 presented with an infection subsequent to colonization (infection group) within 30 days, and 107 showed only intestinal colonization by KpKPC (carrier group). Only the stay in intensive/semi-intensive care units was significantly associated with infection in this cohort (**Table 1**).

**Table 1:** Univariate analysis of risk factors for *K. pneumoniae* infection comparing carriers and patients who developed an infection within 30 days following colonization.

Variable	Carriage (n = 107)	Infection (n = 58)	*P*-value^a^
Age mean (SD)	65 (17.4)	64.9 (17.8)	0.74
Male	69 (65.1)	36 (62.1)	0.735
Diabetes mellitus	14 (13.9)	4 (7.1)	0.297
Chronic renal disease	11 (10.9)	6 (10.7)	1.000
Solid organ transplantation	4(4)	4 (7)	0.457
Onco-hematological disease	2(3.6)	2(2)	0.617
Chemotherapy	2 (2)	1(1.8)	1.000
Carbapenems	51 (50.5)	28 (51.9)	1.000
Quinolones	10 (9.9)	2 (3.7)	0.218
Cephalosporins	35 (34.7)	21 (38.9)	0.604
Hospitalization within the last 2 months	53 (52.5)	34 (61.8)	0.312
Central venous catheter	47(46.5)	27 (48.2)	0.869
Gastrointestinal previous diseases	12 (11.9)	4 (7.1)	0.419
Abdominal surgery	8 (7.9)	6 (10.7)	0.519
Ventilation device	51 (51)	31 (55.4)	0.620
ICU/Semi-intensive care unit (vs. other medical wards)	42 (72.4)	51 (48.1)	0.003

a The P-values for each risk factor were calculated by Fisher’s exact test (categorical variables) or Mann–Whitney test (continuous variables).

**Figure 2:**
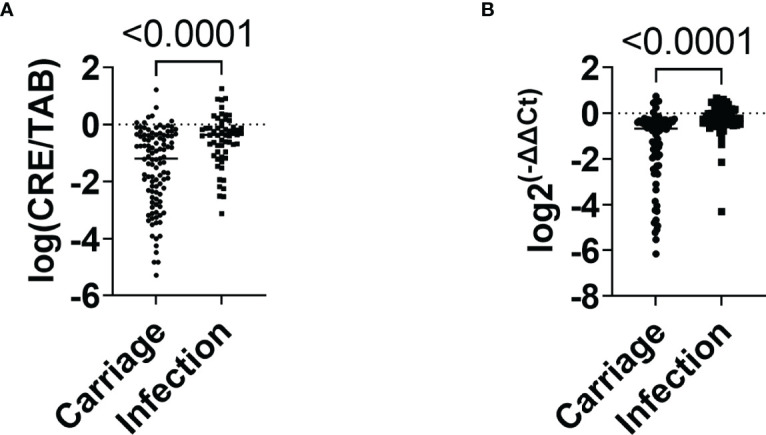
Relative quantification of CRE and *bla*_KPC_-carrying bacteria in carriage and infection groups. **(A)** Relative intestinal load of CRE in carriage and infection groups. **(B)** Relative intestinal loads of bacKPC in carriage and infection groups. *P*-values were calculated using the non-parametric Mann–Whitney test.

The relative intestinal load was determined using two techniques: the microbiological method that measured the load of CRE as related to the total aerobic bacteria present in the sample (results are reported as log CRE/TAB) and the molecular method, comparing *bla*_KPC_ gene copy numbers and the total number of bacteria present in the sample as estimated using primers specific to the conserved region of 16S rRNA gene (results are reported as log 2^-ΔΔCt^). The relative load of CRE in the infection group was higher (median of -0.54, 95% CI: -0.77 to -0.30) than in the carrier group (median of -1.49, 95% CI: -1.76 to -1.23) (*P*<0.0001) (**Figure 2A**). Similarly, the infection group showed a higher relative load of bacKPC from swabs (median of -0.28, 95% CI: -0.51 to -0.06), compared to carriers (median -1.36, 95% CI: -1.69 to -1.02) (*P*<0.0001) (**Figure 2B**).

On the bivariate logistic regression, ICU/semi-intensive care unit admission (OR 2.79, 95% CI 1.375 to 5.687, *P*=0.005), the relative intestinal load of CRE (OR 3.78, 95% CI 1.658 to 8.640, *P*=0.002), and relative intestinal load of bacKPC (OR 8.601, 95% CI 2.44 to 30.352, *P*<0.001), were factors independently associated with infections.

**Figure 3:**
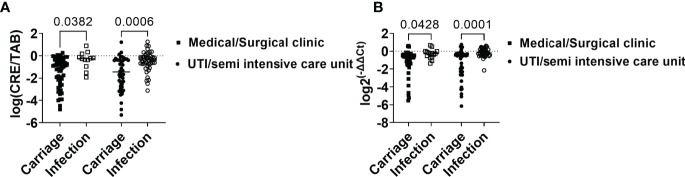
Intestinal load of CRE and *bla*_KPC_-carrying bacteria in association with the infection risk factor “admission to intensive and semi-intensive care units.” **(A)** Relative intestinal loads of CRE in carriage and infection groups considering admission to ICU, semi-intensive care unit, or medical/surgical clinic. **(B)** Relative intestinal loads of bacKPC in carriage and infection groups considering admission to ICU, semi-intensive care unit, or medical/surgical clinic. *P*-values were calculated using the two-way ANOVA test.

However, when analyzed independently, patients from ICU/semi-intensive care units and those admitted to other wards had higher intestinal loads of CRE and bacKPC when they belonged to the infection group when compared with the carriage group (P<0.05; **Figures 3A, B**).

Taken together, these results indicate that patients from the infection group showed higher intestinal loads of CRE and bacKPC when compared with patients who did not present with infection within 30 days of the first positive KpKPC swab.

## Discussion

In Brazil, carbapenem resistance is mostly associated with KPC (Sampaio and Gales, 2016; Ramos-Castaneda et al., 2018). Currently, *K. pneumoniae* carrying KPC is considered epidemic in Brazil and it has been involved in several outbreaks (Campos et al., 2016). Other carbapenemases such as NDM and OXA-48, despite being the main mechanisms that contribute to carbapenem resistance in other countries, are not frequent in Brazil. In agreement with this scenario, in the hospital where the study was carried out, increased rates of carbapenem resistance were accompanied by an increase of KPC detection rates and, during the study period, only 1 isolate of *K. pneumoniae* carrying exclusively *bla*_NDM_ was found and none carrying *bla*_OXA-48_. Thus, due to the importance of KpKPC in the country and in this hospital, our study focused on KpKPC strains.

In this study, colonizing isolates and subsequently infecting isolates from the same patient were highly similar, as assessed by PFGE, corroborating previous studies that evaluated colonizing vs. infecting isolates using MLST, SNP analysis, and cgMLST (Martin et al., 2016).

No specific strain could be associated with transition from colonization to infection, with the most prevalent KpKPC population in each period (i.e. ST437 between 2011 and 2015 and ST11 between 2016 and 2021) being predominantly found associated with both the carriage and infection groups. Although KpKPC ST437 is frequently detected in Brazil, our study and a previous one conducted in another region of Brazil observed ST11 becoming more prevalent after 2014 (Seki et al., 2011; Pereira et al., 2013). *K. pneumoniae* ST11 is considered a global problem due to its broad geographic distribution and antibiotic resistance (Wyres and Holt, 2016). As endemic strains carry virulence and resistance mechanisms, ST11 strains are able to resist antibiotic selective pressure and persist in the clinical environment for years, leading to widespread colonization of patients and subsequent infections (Raro et al., 2020).

A comparison between carriage and infection-related isolates in terms of K- and O-types, the presence of virulence genes such as yersiniabactin and colibactin, or the number of antimicrobial resistance genes, showed no significant differences. These results suggest that a combination of factors, including host and pathogen factors, determine whether carriage develops into infection, as previously proposed (Lewis, 2022).

Intestinal colonization by KpKPC has been related to the development of infections that are difficult to treat (Martin et al., 2016). Prolonged stay in the hospital and the consumption of broad-spectrum antibiotics are the main clinical risk factors related to intestinal colonization by KpKPC due to the disruption of the host’s intestinal homeostasis (Kinashi and Hase, 2021). Most recently, the density of the intestinal load of CRE and bacKPC has been considered a risk factor for the development of infections (Gorrie et al., 2017). Our results are consistent with a previous study performed with 147 adult patients hospitalized in a single health center (Lázaro-Perona et al., 2021). In the study, patients who developed infections showed higher intestinal loads of *K. pneumoniae* harboring *bla*_OXA-48_ (KpOXA) than carriers of KpOXA that had no infections (Lázaro-Perona et al., 2021). Our results add to their observations by reporting that no ST or pulse-type, as determined by the analysis of isolates within a 10-year time line, or resistance and virulence genes showed a significant and independent impact in the transition from colonization to infection by *K. pneumoniae* harboring *bla*_KPC_.

It has been shown that increased levels of *K. pneumoniae* in the intestine are associated with increased inflammation, due to pro-inflammatory cytokines, and decreased the expression of tight-junction-related proteins (e.g., claudin-1, ZO-1, and occludin), increasing the permeability of the intestinal membrane and facilitating the translocation of *K. pneumoniae* to the bloodstream (Lee and Kim, 2011; Joseph et al., 2021). In agreement, prebiotics and probiotics such as *Bifidobacterium bifidum* and *Lactobacillus acidophilus* reduce the expression of inflammatory markers, increase the expression of tight-junction-related proteins, and are also associated with a decrease in the intestinal load of CRE (Marcinkiewicz et al., 2007; Zhou et al., 2009; Ramos-Ramos et al., 2020; Hung et al., 2021). Since CRE carriers contribute to the spread of CRE (Lerner et al., 2015), the use of probiotics can be an important strategy to reduce both the spread of CRE and the development of serious infections during hospitalization (Ramos-Ramos et al., 2020). The use of probiotics in patients colonized by CRE is currently a strategy considered in EUCAST guidelines and should be explored in selected hospitalized patient groups to reduce the intestinal load of pathogenic bacteria (Tacconelli et al., 2019).

Given the difficulty in treating infections caused by Hv and MDR strains, our study and others that address the role of intestinal colonization in infections may support new therapeutic approaches. Since patients admitted to the ICU/semi-intensive care unit have a greater chance of developing infections subsequent to colonization by CRE, the relative quantification of intestinal load may be a useful and cost-effective tool to infer the risk of these patients developing an infection. This practice can contribute to early intervention and reduce the risk of the patient developing a difficult-to-treat infection, thus contributing to a better prognosis and new patient care practices.

## Data Availability Statement

The datasets presented in this study can be found in online repositories. The names of the repository/repositories and accession number(s) can be found in the article/**Supplementary Material**.

## Author Contributions

LM, LL, NC, and MMM: experimental procedures. RS: whole genome assembling and annotation. LM, LL, RS, and PS: data analysis and interpretation. PK and MDVM: isolate maintenance, identification, and antimicrobial susceptibility profiling. AT, FM, and MDVM: epidemiological data analysis and critical review of the paper. LM, LL, and PS: wrote the manuscript with contributions from RS, MMM and JM. JM and PS: conceived the project. PS: supervised the project. All authors contributed to the article and approved the submitted version.

## Funding

This work was supported by Fundaçao de Amparo a Pesquisa do Estado de Sao Paulo (FAPESP) grant 2018/19243-4. LM and RS are supported by a CAPES fellowship and LL by Sociedade Beneficente Israelita Brasileira Albert Einstein.

## Conflict of Interest

The authors declare that the research was conducted in the absence of any commercial or financial relationships that could be construed as a potential conflict of interest.

## Publisher’s Note

All claims expressed in this article are solely those of the authors and do not necessarily represent those of their affiliated organizations, or those of the publisher, the editors and the reviewers. Any product that may be evaluated in this article, or claim that may be made by its manufacturer, is not guaranteed or endorsed by the publisher.
